# Key Considerations From a Health Authority Perspective When Proton Pump Inhibitors Are Used to Treat Gastroesophageal Reflux Disease (GERD) and Their Implications

**DOI:** 10.7759/cureus.31918

**Published:** 2022-11-26

**Authors:** Johanna C Meyer, Sean MacBride-Stewart, Joseph O Fadare, Ammar Abdulrahman Jairoun, Mainul Haque, Amos Massele, Santosh Kumar, Israel Abebrese Sefah, Phumzile P Skosana, Brian Godman

**Affiliations:** 1 Department of Public Health Pharmacy and Management, Sefako Makgatho Health Sciences University, Pretoria, ZAF; 2 Pharmacy Services, Greater Glasgow and Clyde (NHS GGC), Glasgow, GBR; 3 Department of Medicine, Ekiti State University Teaching Hospital, Ado-Ekiti, NGA; 4 Department of Pharmacology and Therapeutics, Ekiti State University, Ado-Ekiti, NGA; 5 Department of Health and Safety, Centre of Medical and Bio allied Health Sciences Research, Ajman University, Dubai, ARE; 6 Department of Pharmacology and Therapeutics, National Defence University of Malaysia, Kuala Lumpur, MYS; 7 Department of Clinical Pharmacology and Therapeutics, Hurbert Kairuki Memorial University, Dar Es Salaam , TZA; 8 Department of Periodontology and Implantology, Karnavati University, Gandhinagar, IND; 9 Department of Pharmacy Practice, School of Pharmacy, University of Health and Allied Sciences, Ho Volta Region, GHA; 10 Department of Public Health Pharmacy and Management, School of Pharmacy, Sefako Makgatho Health Sciences University, Pretoria, ZAF; 11 Centre of Medical and Bio-allied Health Sciences Research, Ajman University, Ajman, ARE; 12 Department of Pharmacoepidemiology, Strathclyde Institute of Pharmacy and Biomedical Sciences, Glasgow, GBR

**Keywords:** hydrogen-potassium adenosine triphosphatase enzyme system antagonist, gastric reflux, thereinafter, connotation, acid reflux, gastroesophageal reflux disease (gerd), therapeutic intervention, proton-pump inhibitors (ppi), public health force viewpoint, principal scrutiny

## Abstract

The growing prevalence of gastroesophageal reflux disease (GERD) needs to be carefully managed to relieve the symptoms and prevent complications. Complications of GERD can include erosive esophagitis, Barrett’s esophagus and gastrointestinal (GI) bleeding. Proton pump inhibitors (PPIs) are typically first-line treatment for GERD alongside lifestyle changes in view of their effectiveness and cost-effectiveness. However, there are concerns with adherence to dosing regimens and recommended lifestyle changes reducing their effectiveness. There are also concerns about potential complications from chronic high-dose PPIs. These include an increased risk of chronic kidney disease, cardiovascular events and infections. Recommendations to physicians include prescribing or dispensing the lowest dose of PPI for the shortest time, with ongoing patient monitoring. Activities among community pharmacists and others have resulted in increased dispensing of PPIs without a prescription, which can be a challenge. PPIs are among the most prescribed and dispensed medicines in view of their effectiveness in managing GERD. However, there are concerns with the doses prescribed and dispensed as well as adherence to lifestyle advice. These issues and challenges need to be addressed by health authorities to maximize the role and value of PPIs.

## Introduction and background

Gastroesophageal reflux disease (GERD) is a digestive disorder resulting from the effortless movement of gastric contents into the esophagus or beyond, which results in troublesome symptoms or more serious complications [1-3]. These include heartburn, a burning sensation in the chest, acid regurgitation, or an ongoing cough [1,3]. Serious complications include dysphagia and Barrett’s esophagus, potentially leading to esophageal adenocarcinoma [3]. GERD is a common condition, often due to abnormalities in the lower esophageal sphincter [4,5], with a pooled global prevalence of 13.3% or more of the population reporting at least weekly symptoms with rates increasing; however, with appreciable geographical variation [1,3,6-10]. Weekly prevalence rates currently range from 2.5% of the population in China up to 51.2% in Greece [6]. Typically, the highest rates are seen among South Asia and Southeast Europe countries at more than 25% of the population, with the lowest rates observed in Canada, France, and Southeast Asia at a prevalence of less than 10% of the population [6]. High prevalence rates are also seen in Nigeria, with prevalence rates increasing [1,11-14]. As a result, GERD is typically the most frequent gastrointestinal disorder across countries [3], with consultation rates in ambulatory care ranging from 5.4% to 56% of all consultations [15,16]. GERD, though cannot be classified as a single disease, is often used as an umbrella term [1,17].

Several lifestyle factors are associated with GERD, including limited exercise, high-fat diets, and alcohol [3,8,13,18,19]. The cornerstone for managing patients with GERD remains lifestyle changes, emphasizing the need to address these factors [1,20-22]. Potential lifestyle modifications include raising the head of the bed, physical exercise including post-dinner walks, reducing smoking and alcohol intake, weight loss in obese patients, avoidance of significant food intake at least two hours before bedtime, especially in patients with nocturnal symptoms, having smaller meals more frequently as opposed to large meals, particularly in the evening, as well as introducing protein-rich meals and a vegetarian diet [8,18,20,23,24]. However, a concern is that lifestyle changes can be overlooked with the increased availability of low-cost treatments, notably in community pharmacies, drug stores, OTC vending machines, and supermarkets, coupled with variable knowledge of GERD among community pharmacists [25-28].

Key points

I. GERD is a common condition across countries, with prevalence rates continuing to grow.

II. Optimal management includes lifestyle advice and medical management, which is increasingly PPIs. The combined approach can help enhance the quality of life of patients as well as reduce potential complications of erosive esophagitis and Barrett’s esophagus.

III. There are concerns with chronic PPI use, especially with high doses, as this can lead to complications unless addressed with key concerns, including potentially enhancing polypharmacy and chronic kidney diseases, fractures, and infections as well as potentially enhancing polypharmacy. However, this must be balanced against their undoubted effectiveness.

IV. Potential concerns can be addressed by only prescribing or dispensing PPIs for relevant indications, providing advice on the optimal timing for administration, and regularly monitoring patients. Alongside this, encouraging adherence to lifestyle advice.

V. Community pharmacists and others are well-placed to monitor patients and offer advice. Consequently, there can be concerns with OTC vending machines containing PPIs unless addressed; however, this may be less of an issue with doses of PPIs available as OTC pharmaceutical products.

## Review

Medical management of GERD, including activities of health authorities

GERD needs to be actively managed to prevent complications, which can arise from the disease itself, in addition to heartburn and associated pain, subsequently affecting patients’ quality of life [12,29]. Dominant complications of GERD include dysphagia, with an appreciable number of patients with dysphagia having acid-related disorders [3,30] and bleeding from erosive esophagitis. The prevalence of erosive esophagitis ranges from 6.4% in China up to 15.5% in Sweden in patients with symptoms of GERD [3,31,32]. Approximately one-quarter of non-erosive reflux disease (NERD) patients have erosive esophagitis on repeat endoscopy two years later [3]. This is a concern since between 2003 and 2006, in the USA, there were approximately 10,570 hospital admissions annually due to erosive esophagitis [3,33]. Barrett’s esophagus is also a complication with GERD following on from NERD and dysphagia, with some studies suggesting erosive esophagitis as a major risk factor for Barrett’s esophagus, with up to a fivefold increased risk [9,34,35]. The risk of Barrett’s esophagus increases with the length of time of untreated GERD [36] and is typically a precursor to esophageal adenocarcinoma [3,35,37]. Eusebi et al. recently estimated the pooled prevalence of patients with confirmed Barrett’s esophagus in those with GERD at 7.0%, higher among those who drank alcohol [17].

Antacids, alginates, histamine (H_2_) receptor blockers, or proton pump inhibitors (PPIs) are typically a first-line medical treatment for patients with GERD [1,38-40]. In recent years, PPIs, e.g., omeprazole, which work by covalently binding to the proton pumps that control the final step of gastric acid production, thereby decreasing it, have become the treatment of choice for patients with GERD, with a number of studies showing superiority over H_2_ receptor blockers [5,38,41-49]. Typically, a standard dose of a PPI provides complete relief in approximately 70 to 80% of patients with GERD within a week [1,50-53], with limited differences in effectiveness between twice-daily and once-daily PPIs [54]. However, some studies have suggested otherwise [55].

**Figure 1:**
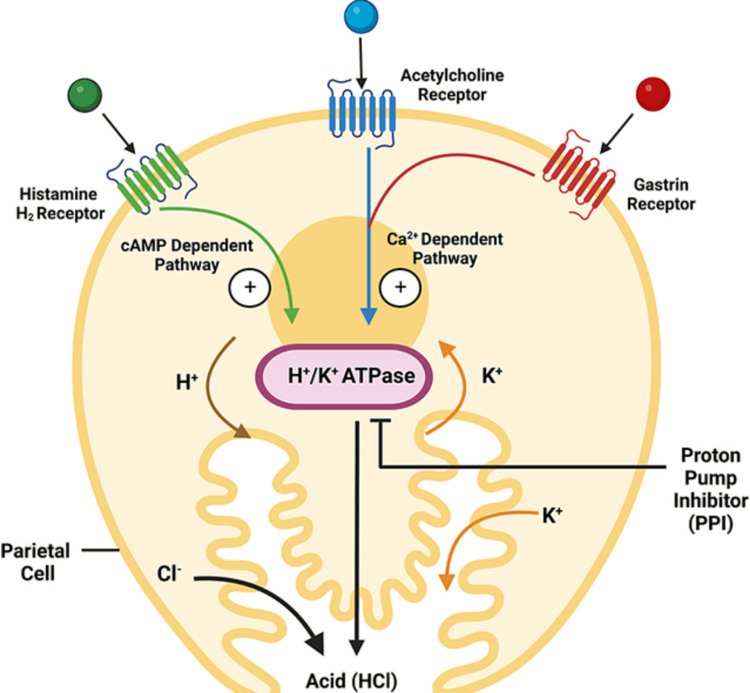
Diagrammatic presentation of inhibition of gastric HCl secretion by proton pump inhibitors (PPIs). This figure has been developed using the premium version of Biorender [https://biorender.com/] with the license number: TE24NLD7LY. Image Credit: Susmita Sinha.

A double dose of PPIs can also potentially be administered in patients with GERD not responding to a single dose [1,47,56,57]. The long-term use of double-dose PPIs has been found to be safe; however, their prolonged use should be limited to situations where the benefits outweigh the risks [5,58-60]. In addition, patients are regularly reviewed [5,60]. Alternatively, prokinetics can be added to PPIs to improve their effectiveness (Figure 1) [61].

Despite their undoubted effectiveness, compliance with PPIs is important for the optimal management of patients with GERD [20]. This should be combined with compliance with counseling on lifestyle changes [62], with weight loss potentially reducing PPI doses [22]. Overall, optimal dosing of PPIs combined with lifestyle management appears to be the most effective and cost-effective approach to managing patients with GERD where lifestyle changes are insufficient [1,5,20,44].

Compliance with PPIs can, however, be of particular concern if patients obtain these medicines over the counter (OTC) or via OTC vending machines without support and advice from healthcare professionals (HCPs), including community pharmacists [20,63]. This is because studies have shown that if patients are not fully compliant with taking their PPIs, i.e., not consuming certain PPIs, i.e., not consuming medication 30 minutes prior to a meal, they can become refractory and cause adverse impacts to patients [64]. Overall, 40% to 50% of patients do not comply with optimal timing, and poor compliance is an important reason for PPI failure [44,57]. Concerns are enhanced by the fact that compliance with PPI dosing regimens generally decreases over time, although compliance rates are higher in patients with Barrett’s esophagus [65]. PPIs taken before supper appear to be the most effective [66], with the potential monitoring of stomach pH levels to detect non-responders [67]. Although monitoring the stomach pH levels is considered the model diagnostic procedure to obtain outstanding clinical outcomes among PPI recipients. However, surveillance of pH in ambulatory care is often inconvenient, cumbersome, and expensive, impacting routine use [4, 68,69].

More countries are making PPIs available OTC, which will continue [70]. Community pharmacists can play a key role in identifying patients with appropriate symptoms of GERD as well as improving medication compliance, with the use of OTC PPIs increasing due to the convenience of community pharmacies, negating the need for patients to consult a physician with their symptoms with potentially increased costs [15,53,71,72]. In identifying patients with GERD, community pharmacists must be skilled in good history-taking regarding the patient’s symptoms, medication use, lifestyle, and communication. This will ensure that they ask the correct questions to be able to make an informed decision and recommend the correct product [25]. Community pharmacists can also suggest referrals to a physician where there are concerns, which include multiple episodes of GERD per week, persistent or recurrent symptoms after a month of taking OTC PPIs, or unintentional weight loss [53,73,74]. This builds on multiple studies demonstrating improvements in patient care following input from community pharmacists across disease areas [75-77].

With PPIs firmly established as a first-line pharmacologic treatment for patients with GERD [1,5,20,44,47], which PPI to prescribe should be based on key issues, including their effectiveness, safety, and cost-effectiveness as there can be appreciable cost variations between the different PPIs [55]. In their review, Graham and Tansel (2018) and others have concluded that all PPIs are functionally equivalent [55,78]. This echoed the beliefs among health authorities across Western Europe when generic omeprazole first became available at appreciably lower prices than patented PPIs [79-82]. Multiple demand-side measures introduced by several health authorities, alongside measures to appreciably lower the prices of multiple-sourced PPIs, resulted in low overall expenditure on PPIs in the Netherlands, Sweden, and the UK, despite appreciably increased utilization [81-86]. In Scotland, combined supply- and demand-side measures decreased PPI expenditure by 67% between 2001 and 2016 despite a three-fold increase in utilization with generic PPIs priced as low as 8.5% of pre-patent loss prices [83]. A similar picture was seen in Sweden with expenditure on PPIs, adjusted for population size, one-tenth of that seen in Ireland in 2007 with its limited demand-side measures encouraging the preferential prescribing of low-cost multiple-sourced PPIs [81,84]. Three-monthly contracting in the Netherlands resulted in prices of generic omeprazole being as low as 2% of pre-patent loss prices, which resulted in considerable savings [82].

Low prices for PPIs are important in countries given appreciably increased utilization over the last 10 to 20 years, especially countries with universal healthcare and competing demands on available resources, with published studies showing no difference in the effectiveness and safety of generic versus originator PPIs, like other disease areas [87-92]. Low-cost multiple-sourced PPIs are also essential for patients with GERD in low- and middle-income countries (LMICs) with their high patient co-payment levels. When family members become ill, this can have catastrophic consequences for the rest of the family [92-95]. The availability of low-cost generic PPIs in several Central and Eastern European countries has also enhanced their use following the easing of prescribing restrictions [96,97]. However, there have been concerns with the quality of generics in some LMICs, which need to be addressed, to enhance the use of multiple-sourced PPIs in patients with GERD [84,98]. According to the WHO, as many as one in 10 medicines sold in low- and middle-income countries are either substandard or falsified. Initiatives such as the Lomé initiative for counterfeit medicines can help here alongside health authorities and community pharmacies regularly checking the packaging and content of PPIs supplied, including OTC PPIs [99], building on similar initiatives with herbal medicines [100]. There can, though, be concerns with the level of checking among community pharmacists in some LMICs [101,102]. 

**Figure 2:**
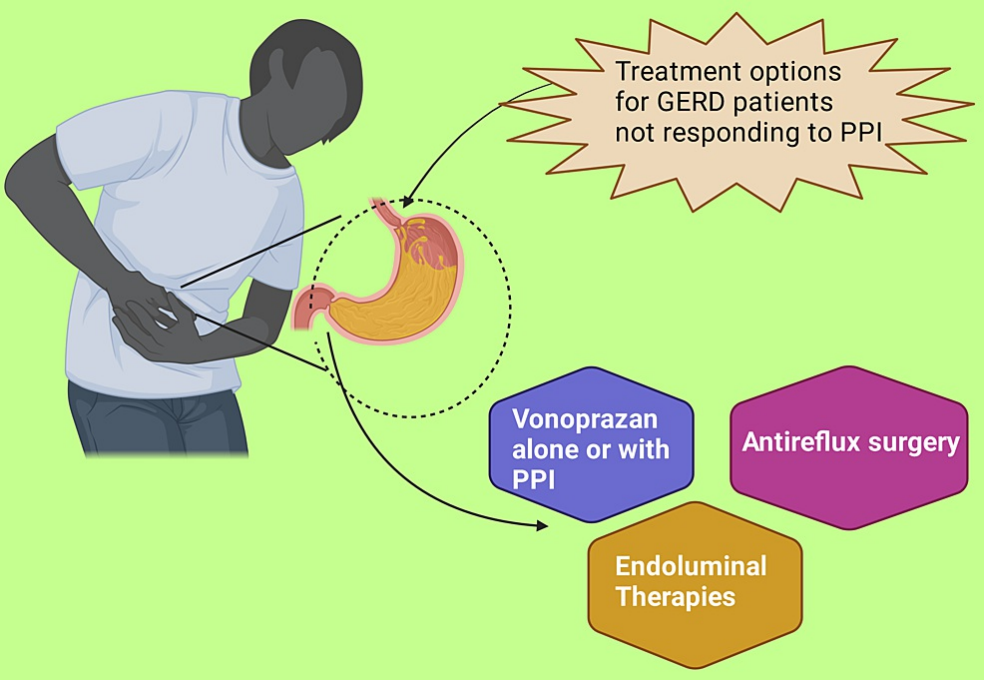
Illustrating treatment options for GERD not responding to PPI. PPI: Proton pump Inhibitor. This figure has been created using the premium version of Biorender (www.biorender.com) with the license number: ZJ24NL4X09. Image Credit: Rahnuma Ahmad.

Despite their undoubted effectiveness, up to 30% of patients with GERD have persistent symptoms, and up to 44% have partial or no response to PPIs [51]. This is a concern as a poor response will have an impact on the patient's physical and mental health [51]. Treatment options at this stage include vonoprazan, a potassium-competitive acid blocker, either alone or with PPIs [1,103,104]. However, longer-term studies are needed to fully evaluate its place in treatment, especially with the availability of low-cost multiple-sourced PPIs [1]. Anti-reflux surgery is another option in pertinent patients [1,20,105,106]. PPIs may, though, still be needed after surgery to improve patient-relevant outcomes [20,107]. Endoluminal therapies are also increasingly being used for managing patients with refractory GERD (Figure 2) [20,47,108]. These approaches, though, are outside the scope of this paper.

In addition to these issues, there are concerns with the long-term complications of PPI use and the potential impact on enhancing polypharmacy and associated matters. However, these concerns must be balanced against their undoubted effectiveness especially given issues with the complications of GERD as well as patients still being admitted to hospitals with stomach ulcers [5,83]. In addition, these issues and concerns may be less of a problem at doses of PPIs available OTC [74].

Potential overuse of PPIs and the implications

The practice of self-medication is common worldwide, including in LMICs and high-income countries, and may even be more common than prescribed medicines, especially in LMICs [109,110]. OTC medicines provide symptomatic relief and a solution to individuals having immediate health problems, as well as lower costs for patients where there are high co-payments for physician visits and travel costs, which can be catastrophic for some families [15,72,95,111]. However, the inappropriate use of OTC medicines can have potential dangers, including incorrect self-diagnosis, adverse events and hazardous drug-drug interactions, incorrect choice of therapy and dosing, and masking severe disease [109,112-114].

The high prevalence of GERD, the effectiveness of PPIs versus antacids and H_2 _receptor blockers, coupled with their low prices with multiple sources now available, has resulted in PPIs becoming one of the most utilized medicines across countries, especially among high-income countries [115-117]. Other factors increasing PPI use include their increased prescribing as prophylaxis for elderly patients with arthritis as well as those on multiple medications. In addition, patients are being prescribed PPIs alongside anti-coagulant therapy to reduce potential GI bleeding and avoidable hospital admissions [83,118-121]. However, these combined factors have resulted in the potential overuse of PPIs leading to possible complications [83,117,118]. Mares-Garcia et al. found that over a third of patients over the age of 60 years had no obvious reason for being prescribed a PPI [116], which needs to be addressed.

The overuse of PPIs can increase polypharmacy, especially in the elderly, with challenges of adherence to prescribed medicines alongside increasing adverse drug reactions (ADRs) [122,123], which is a concern for health authorities. ADRs can be problematic as they increase morbidity, mortality, and costs [124-126]. There is also an increased risk of chronic kidney disease and fragility fractures in patients taking PPIs long-term, resulting in advice from HCPs and health authorities for patients to increase their calcium and Vitamin D intake, especially among the elderly [83,127-130]. However, other studies have failed to show an association between long-term PPI use and changes in bone mineral density [59,131].

Increased counseling by community pharmacists, helped by training, alongside increased patient education, can help address polypharmacy issues as well as adherence concerns when combined with general discussions on the patient’s symptoms to direct appropriate care [25,55,132]. Patients on chronic PPIs should also ideally have their serum magnesium levels regularly checked if they are also taking medicines known to cause hypomagnesemia [130,133]. Again, this can be difficult for patients to obtain their PPIs from pharmacies and other outlets, including OTC vending machines, as well as among ambulatory care physicians, unless there are recall systems in place to regularly undertake medication reviews. This, though, may again be less of an issue when patients are using PPIs for a short duration.

There may also be an increased risk of cardiovascular and cerebrovascular events linked with chronic PPI treatment, especially among patients prescribed anti-platelet medicines; however, there is conflicting evidence [134-138]. Prescribers may want to keep this in mind when managing patients with GERD and cardiovascular diseases, and pharmacists and their assistants when dispensing PPIs. Alongside this, there can be an increased risk of small intestinal bacterial overgrowth as well as enteric infections with chronic PPIs [139-143]. This was a concern during the recent COVID-19 pandemic, with studies suggesting a dose-response relationship between PPIs and COVID-19, with patients taking lower-dose PPIs appearing at reduced risk of COVID-19 [139]. However, there have been concerns that these findings were based on a cross-sectional study [139]. In any event, this may now be less of an issue with the availability of effective vaccines against COVID-19 [144,145].

Concerns with the potential adverse effects of chronic PPI use have resulted in guidance from health authorities across countries, including Scotland, to prescribe the lowest possible dose and regularly review patients on long-term PPIs [16,83,143,146]. Alongside this, regularly monitor prescribed doses of PPIs [16,83,147]. This builds on general advice in Scotland over several years to reduce the doses of PPIs prescribed for patients on chronic medication as well as monitor general prescribing [83,146]. However, these concerns and issues with the current overuse of PPIs, especially in high-income countries, need to be balanced against the undoubted effectiveness of PPIs in preventing and healing ulcers, controlling symptoms of GERD, and limiting the progress of patients toward erosive esophagitis and potentially Barrett’s esophagus with its associated implications. In addition, reducing hospital admissions arising from GI bleeds.

Next steps for better clinical outcome

PPIs should be prescribed for GERD only when clinically indicated, in a brief course, and in the smallest possible dose. PPIs receiver should be regularly monitored, especially those at increased risk of complications and taking multiple medications [117,139]. In addition, health authorities must keep an eye on physician compliance with agreed guidelines, with subsequent compliance to guidelines improving future care [115,148-150]. Additionally, there are reports that PPIs are often obtained or procured from OTC vending machines and non-pharmacy stores without appropriate medical prescribing advice. These apprehensions are possible to minimize with the low doses of PPIs available in OTC and OTC vending machines helping with testing and social distancing during pandemics [27,151]. Heath authorities must keep track of pharmacy shops to stop selling PPIs without a prescription, including self-medications.

## Conclusions

There is a growing use of PPIs across countries to manage patients with GERD. However, there are concerns with their chronic use as well as with compliance and adherence rates in practice. This has resulted in advice from health authorities and others to prescribe PPIs Together with this, HCPs need to enhance patient compliance to PPIs and their dosage regimen, as well as other prescribed medicines, alongside agreed changes in their lifestyle, to help alleviate their symptoms and reduce future complications.

Community pharmacists are well-positioned to ensure that PPIs are used appropriately and effectively while also working with patients to reduce their overuse of PPIs. Pharmacists can provide effective counseling to help ensure PPI use is for agreed indications with the lowest effective dose dispensed for the shortest time. Complementary to this, conveying the importance of adherence to lifestyle changes as well as suggested dosing regimens for PPIs to improve symptom control and optimize the likelihood of treatment success. Consequently, PPIs should only be available as prescribed and health authorities should implement stringent policy monitoring. We will be following this up in future research. These suggestions and activities will continually be monitored by health authorities, especially with increasing rates of GERD with rising obesity and elderly rates across countries.

## References

[1] Jung HK, Tae CH, Song KH (2021). 2020 Seoul consensus on the diagnosis and management of gastroesophageal reflux disease. J Neurogastroenterol Motil.

[2] Vakil N, van Zanten SV, Kahrilas P, Dent J, Jones R (2006). The Montreal definition and classification of gastroesophageal reflux disease: a global evidence-based consensus. Am J Gastroenterol.

[3] Richter JE, Rubenstein JH (2018). Presentation and epidemiology of gastroesophageal reflux disease. Gastroenterology.

[4] Clarrett DM, Hachem C (2018). Gastroesophageal reflux disease (GERD). Mo Med.

[5] Turshudzhyan A, Samuel S, Tawfik A, Tadros M (2022). Rebuilding trust in proton pump inhibitor therapy. World J Gastroenterol.

[6] Eusebi LH, Ratnakumaran R, Yuan Y, Solaymani-Dodaran M, Bazzoli F, Ford AC (2018). Global prevalence of, and risk factors for, gastro-oesophageal reflux symptoms: a meta-analysis. Gut.

[7] Karimian M, Nourmohammadi H, Salamati M, Hafezi Ahmadi MR, Kazemi F, Azami M (2020). Epidemiology of gastroesophageal reflux disease in Iran: a systematic review and meta-analysis. BMC Gastroenterol.

[8] Zhang M, Hou ZK, Huang ZB, Chen XL, Liu FB (2021). Dietary and lifestyle factors related to gastroesophageal reflux disease: a systematic review. Ther Clin Risk Manag.

[9] Asreah RH, Abdullhameed A (2021). Risk factors of erosive esophagitis and barrett's esophagus in patients with reflux symptoms. Med J Islam Repub Iran.

[10] Abrahami D, McDonald EG, Schnitzer M, Azoulay L (2021). Trends in prescribing patterns of proton pump inhibitors surrounding new guidelines. Ann Epidemiol.

[11] Akinola MA, Oyedele TA, Akande KO, Oluyemi OY, Salami OF, Adesina AM, Adebajo AD (2020). Gastroesophageal reflux disease: prevalence and Extraesophageal manifestations among undergraduate students in South West Nigeria. BMC Gastroenterol.

[12] Nwokediuko SC, Adekanle O, Akere A (2020). Gastroesophageal reflux disease in a typical African population: a symptom-based multicenter study. BMC Gastroenterol.

[13] Jemilohun AC, Oyelade BO, Fadare JO, Amole IO (2018). Gastroesophageal reflux disease and etiological correlates among nigerian adults at ogbomoso. Ann Ib Postgrad Med.

[14] Maiyaki A, Borodo M, Samaila A, Yakubu A (2018). Prevalence of gastroesophageal reflux disease among patients with dyspepsia undergoing endoscopy in a tertiary hospital in Nigeria. Sahel Med J.

[15] Hungin AP, Hill C, Raghunath A (2009). Systematic review: frequency and reasons for consultation for gastro-oesophageal reflux disease and dyspepsia. Aliment Pharmacol Ther.

[16] (2014). National Therapeutic Indicators 2014/2015. http://www.sehd.scot.nhs.uk/publications/DC20141201nti.pdf.

[17] Eusebi LH, Telese A, Cirota GG, Haidry R, Zagari RM, Bazzoli F, Ford AC (2021). Systematic review with meta-analysis: risk factors for Barrett's oesophagus in individuals with gastro-oesophageal reflux symptoms. Aliment Pharmacol Ther.

[18] Ahmed S, Jamil S, Shaikh H, Abbasi M (2020). Effects of Life style factors on the symptoms of gastro esophageal reflux disease: a cross sectional study in a Pakistani population. Pak J Med Sci.

[19] Heidarzadeh-Esfahani N, Soleimani D, Hajiahmadi S, Moradi S, Heidarzadeh N, Nachvak SM (2021). Dietary intake in relation to the risk of reflux disease: a systematic review. Prev Nutr Food Sci.

[20] Sandhu DS, Fass R (2018). Current trends in the management of gastroesophageal reflux disease. Gut Liver.

[21] Singh M, Lee J, Gupta N (2013). Weight loss can lead to resolution of gastroesophageal reflux disease symptoms: a prospective intervention trial. Obesity (Silver Spring).

[22] de Bortoli N, Guidi G, Martinucci I (2016). Voluntary and controlled weight loss can reduce symptoms and proton pump inhibitor use and dosage in patients with gastroesophageal reflux disease: a comparative study. Dis Esophagus.

[23] Albarqouni L, Moynihan R, Clark J, Scott AM, Duggan A, Del Mar C (2021). Head of bed elevation to relieve gastroesophageal reflux symptoms: a systematic review. BMC Fam Pract.

[24] Jacobson BC, Somers SC, Fuchs CS, Kelly CP, Camargo CA Jr (2006). Body-mass index and symptoms of gastroesophageal reflux in women. N Engl J Med.

[25] Showande SJ, Adelakun AR (2019). Management of uncomplicated gastric ulcer in community pharmacy: a pseudo-patient study. Int J Clin Pharm.

[26] Collins S (2022). More states allow sales of OTCs in vending machines. Pharmacy Today.

[27] Jairoun AA, Al Hemyari SS, Abdulla NM (2022). Acceptability and willingness of UAE residents to use OTC vending machines to deliver self-testing kits for COVID-19 and the implications. J Multidiscip Healthc.

[28] Singer J (2020). Bring drug dispensing into the modern age with vending machines. https://www.acsh.org/news/2020/03/10/bring-drug-dispensing-modern-age-vending-machines-14627.

[29] Tack J, Becher A, Mulligan C, Johnson DA (2012). Systematic review: the burden of disruptive gastro-oesophageal reflux disease on health-related quality of life. Aliment Pharmacol Ther.

[30] Howden CW (2004). Management of acid-related disorders in patients with dysphagia. Am J Med.

[31] Dent J, Becher A, Sung J, Zou D, Agréus L, Bazzoli F (2012). Systematic review: patterns of reflux-induced symptoms and esophageal endoscopic findings in large-scale surveys. Clin Gastroenterol Hepatol.

[32] Zou D, He J, Ma X (2011). Epidemiology of symptom-defined gastroesophageal reflux disease and reflux esophagitis: the systematic investigation of gastrointestinal diseases in China (SILC). Scand J Gastroenterol.

[33] Thukkani N, Sonnenberg A (2010). The influence of environmental risk factors in hospitalization for gastro-oesophageal reflux disease-related diagnoses in the United States. Aliment Pharmacol Ther.

[34] Ronkainen J, Talley NJ, Storskrubb T (2011). Erosive esophagitis is a risk factor for Barrett's esophagus: a community-based endoscopic follow-up study. Am J Gastroenterol.

[35] Zerbib F (2018). Erosive esophagitis. Gastrointestinal Motility Disorders.

[36] Sharma N, Ho KY (2016). Risk factors for Barrett's oesophagus. Gastrointest Tumors.

[37] Jain S, Dhingra S (2017). Pathology of esophageal cancer and Barrett's esophagus. Ann Cardiothorac Surg.

[38] Hosseini M, Salari R, Shariatmaghani S, Birjandi B, Salari M (2017). Gastrointestinal symptoms associated with gastroesophageal reflux disease, and their relapses after treatment with proton pump inhibitors: a systematic review. Electron Physician.

[39] Zhao CX, Wang JW, Gong M (2020). Efficacy and safety of alginate formulations in patients with gastroesophageal reflux disease: a systematic review and meta-analysis of randomized controlled trials. Eur Rev Med Pharmacol Sci.

[40] Kwiatek MA, Roman S, Fareeduddin A, Pandolfino JE, Kahrilas PJ (2011). An alginate-antacid formulation (Gaviscon Double Action Liquid) can eliminate or displace the postprandial 'acid pocket' in symptomatic GERD patients. Aliment Pharmacol Ther.

[41] Chiba N, De Gara CJ, Wilkinson JM, Hunt RH (1997). Speed of healing and symptom relief in grade II to IV gastroesophageal reflux disease: a meta-analysis. Gastroenterology.

[42] Armstrong D, Paré P, Pericak D, Pyzyk M; Canadian Pantoprazole GERD Study Group (2001). Symptom relief in gastroesophageal reflux disease: a randomized, controlled comparison of pantoprazole and nizatidine in a mixed patient population with erosive esophagitis or endoscopy-negative reflux disease. Am J Gastroenterol.

[43] Sigterman KE, van Pinxteren B, Bonis PA, Lau J, Numans ME (2013). Short-term treatment with proton pump inhibitors, H2-receptor antagonists and prokinetics for gastro-oesophageal reflux disease-like symptoms and endoscopy negative reflux disease. Cochrane Database Syst Rev.

[44] Strand DS, Kim D, Peura DA (2017). 25 years of proton pump inhibitors: a comprehensive review. Gut Liver.

[45] Fass R, Inadomi J, Han C, Mody R, O'Neil J, Perez MC (2012). Maintenance of heartburn relief after step-down from twice-daily proton pump inhibitor to once-daily dexlansoprazole modified release. Clin Gastroenterol Hepatol.

[46] Vigneri S, Termini R, Leandro G (1995). A comparison of five maintenance therapies for reflux esophagitis. N Engl J Med.

[47] Gyawali CP, Fass R (2018). Management of gastroesophageal reflux disease. Gastroenterology.

[48] Ahmed A, Clarke JO (2022). Proton Pump Inhibitors (PPI). https://www.ncbi.nlm.nih.gov/books/NBK557385/.

[49] Schubert ML (2010). Gastric secretion. Curr Opin Gastroenterol.

[50] Katz PO, Gerson LB, Vela MF (2013). Guidelines for the diagnosis and management of gastroesophageal reflux disease. Am J Gastroenterol.

[51] Becher A, El-Serag H (2011). Systematic review: the association between symptomatic response to proton pump inhibitors and health-related quality of life in patients with gastro-oesophageal reflux disease. Aliment Pharmacol Ther.

[52] El-Serag H, Becher A, Jones R (2010). Systematic review: persistent reflux symptoms on proton pump inhibitor therapy in primary care and community studies. Aliment Pharmacol Ther.

[53] Boardman HF, Heeley G (2015). The role of the pharmacist in the selection and use of over-the-counter proton-pump inhibitors. Int J Clin Pharm.

[54] Zhang H, Yang Z, Ni Z, Shi Y (2017). A meta-analysis and systematic review of the efficacy of twice daily PPIs versus once daily for treatment of gastroesophageal reflux disease. Gastroenterol Res Pract.

[55] Graham DY, Tansel A (2018). Interchangeable use of proton pump inhibitors based on relative potency. Clin Gastroenterol Hepatol.

[56] Kinoshita Y, Kato M, Fujishiro M (2018). Efficacy and safety of twice-daily rabeprazole maintenance therapy for patients with reflux esophagitis refractory to standard once-daily proton pump inhibitor: the Japan-based EXTEND study. J Gastroenterol.

[57] Domingues G, Moraes-Filho JP (2014). Noncompliance is an impact factor in the treatment of gastroesophageal reflux disease. Expert Rev Gastroenterol Hepatol.

[58] Torvinen-Kiiskinen S, Tolppanen AM, Koponen M, Tanskanen A, Tiihonen J, Hartikainen S, Taipale H (2018). Proton pump inhibitor use and risk of hip fractures among community-dwelling persons with Alzheimer's disease-a nested case-control study. Aliment Pharmacol Ther.

[59] Targownik LE, Goertzen AL, Luo Y, Leslie WD (2017). Long-term proton pump inhibitor use is not associated with changes in bone strength and structure. Am J Gastroenterol.

[60] Targownik LE, Fisher DA, Saini SD (2022). Aga clinical practice update on de-prescribing of proton pump inhibitors: expert review. Gastroenterology.

[61] Jung DH, Huh CW, Lee SK, Park JC, Shin SK, Lee YC (2021). A systematic review and meta-analysis of randomized control trials: combination treatment with proton pump inhibitor plus prokinetic for gastroesophageal reflux disease. J Neurogastroenterol Motil.

[62] Kaltenbach T, Crockett S, Gerson LB (2006). Are lifestyle measures effective in patients with gastroesophageal reflux disease? An evidence-based approach. Arch Intern Med.

[63] Sheikh I, Waghray A, Waghray N, Dong C, Wolfe MM (2014). Consumer use of over-the-counter proton pump inhibitors in patients with gastroesophageal reflux disease. Am J Gastroenterol.

[64] Gunaratnam NT, Jessup TP, Inadomi J, Lascewski DP (2006). Sub-optimal proton pump inhibitor dosing is prevalent in patients with poorly controlled gastro-oesophageal reflux disease. Aliment Pharmacol Ther.

[65] El-Serag HB, Fitzgerald S, Richardson P (2009). The extent and determinants of prescribing and adherence with acid-reducing medications: a national claims database study. Am J Gastroenterol.

[66] Hatlebakk JG, Katz PO, Kuo B, Castell DO (1998). Nocturnal gastric acidity and acid breakthrough on different regimens of omeprazole 40 mg daily. Aliment Pharmacol Ther.

[67] Gomollón F, Calvet X (2005). Optimising acid inhibition treatment. Drugs.

[68] Badillo R, Francis D (2014). Diagnosis and treatment of gastroesophageal reflux disease. World J Gastrointest Pharmacol Ther.

[69] Gyawali CP, Kahrilas PJ, Savarino E (2018). Modern diagnosis of GERD: the Lyon consensus. Gut.

[70] Oleszkiewicz P, Krysinski J, Religioni U, Merks P (2021). Access to medicines via non-pharmacy outlets in European countries-a review of regulations and the influence on the self-medication phenomenon. Healthcare (Basel).

[71] Akinyinka Akinyinka, Adebayo BI, Wright K, Adeniran A (2016). Client waiting time in an urban primary health care centre in Lagos. J Com Med Primary Health Care.

[72] Haseeb A, Bilal M (2016). Prevalence of using non prescribed medications in economically deprived rural population of Pakistan. Arch Public Health.

[73] Holtmann G, Bigard MA, Malfertheiner P, Pounder R (2011). Guidance on the use of over-the-counter proton pump inhibitors for the treatment of GERD. Int J Clin Pharm.

[74] Johnson DA, Katz PO, Armstrong D (2017). The safety of appropriate use of over-the-counter proton pump inhibitors: an evidence-based review and Delphi consensus. Drugs.

[75] Abdulsalim S, Unnikrishnan MK, Manu MK, Alrasheedy AA, Godman B, Morisky DE (2018). Structured pharmacist-led intervention programme to improve medication adherence in COPD patients: a randomized controlled study. Res Social Adm Pharm.

[76] Rajiah K, Sivarasa S, Maharajan MK (2021). Impact of pharmacists’ interventions and patients’ decision on health outcomes in terms of medication adherence and quality use of medicines among patients attending community pharmacies: a systematic review. Int J Environ Res Public Health.

[77] Milosavljevic A, Aspden T, Harrison J (2018). Community pharmacist-led interventions and their impact on patients' medication adherence and other health outcomes: a systematic review. Int J Pharm Pract.

[78] Kaniecki T, Abdi T, McMahan ZH (2021). A practical approach to the evaluation and management of gastrointestinal symptoms in patients with systemic sclerosis. Best Pract Res Clin Rheumatol.

[79] Fraeyman J, Van Hal G, Godman B, Beutels P (2013). The potential influence of various initiatives to improve rational prescribing for proton pump inhibitors and statins in Belgium. Expert Rev Pharmacoecon Outcomes Res.

[80] Godman B, Wettermark B, van Woerkom M (2014). Multiple policies to enhance prescribing efficiency for established medicines in Europe with a particular focus on demand-side measures: findings and future implications. Front Pharmacol.

[81] Godman B, Shrank W, Andersen M (2010). Comparing policies to enhance prescribing efficiency in Europe through increasing generic utilization: changes seen and global implications. Expert Rev Pharmacoecon Outcomes Res.

[82] Woerkom MV, Piepenbrink H, Godman B (2012). Ongoing measures to enhance the efficiency of prescribing of proton pump inhibitors and statins in The Netherlands: influence and future implications. J Comp Eff Res.

[83] Godman B, Kurdi A, McCabe H (2018). Ongoing activities to influence the prescribing of proton pump inhibitors within the Scottish National Health Service: their effect and implications. Gener Biosimilars Initiative J.

[84] Godman B, Fadare J, Kwon HY (2021). Evidence-based public policy making for medicines across countries: findings and implications for the future. J Comp Eff Res.

[85] Godman B, Wettermark B, Hoffmann M, Andersson K, Haycox A, Gustafsson LL (2009). Multifaceted national and regional drug reforms and initiatives in ambulatory care in Sweden: global relevance. Expert Rev Pharmacoecon Outcomes Res.

[86] McGinn D, Godman B, Lonsdale J, Way R, Wettermark B, Haycox A (2010). Initiatives to enhance the quality and efficiency of statin and PPI prescribing in the UK: impact and implications. Expert Rev Pharmacoecon Outcomes Res.

[87] Dackus GH, Loffeld SM, Loffeld RJ (2012). Use of acid suppressive therapy more than 10 years after the endoscopic diagnosis of reflux esophagitis with specific emphasis to trademark and generic proton pump inhibitors. J Gastroenterol Hepatol.

[88] Corrao G, Soranna D, Merlino L, Mancia G (2014). Similarity between generic and brand-name antihypertensive drugs for primary prevention of cardiovascular disease: evidence from a large population-based study. Eur J Clin Invest.

[89] Desai RJ, Sarpatwari A, Dejene S (2019). Comparative effectiveness of generic and brand-name medication use: a database study of US health insurance claims. PLoS Med.

[90] Manzoli L, Flacco ME, Boccia S (2016). Generic versus brand-name drugs used in cardiovascular diseases. Eur J Epidemiol.

[91] Lin YS, Jan IS, Cheng SH (2017). Comparative analysis of the cost and effectiveness of generic and brand-name antibiotics: the case of uncomplicated urinary tract infection. Pharmacoepidemiol Drug Saf.

[92] Godman B, Massele A, Fadare J (2021). Generic drugs - essential for the sustainability of healthcare systems with numerous strategies to enhance their use. Pharm Sci Biomed Anal J.

[93] Cameron A, Ewen M, Ross-Degnan D, Ball D, Laing R (2009). Medicine prices, availability, and affordability in 36 developing and middle-income countries: a secondary analysis. Lancet.

[94] Kastor A, Mohanty SK (2018). Disease-specific out-of-pocket and catastrophic health expenditure on hospitalization in India: do Indian households face distress health financing?. PLoS One.

[95] Aregbeshola BS, Khan SM (2018). Out-of-pocket payments, catastrophic health expenditure and poverty among households in Nigeria 2010. Int J Health Policy Manag.

[96] Garuolienė K, Godman B, Gulbinovič J, Schiffers K, Wettermark B (2016). Differences in utilization rates between commercial and administrative databases: implications for future health-economic and cross-national studies. Expert Rev Pharmacoecon Outcomes Res.

[97] Markovic-Pekovic V, Skrbić R, Godman B, Gustafsson LL (2012). Ongoing initiatives in the Republic of Srpska to enhance prescribing efficiency: influence and future directions. Expert Rev Pharmacoecon Outcomes Res.

[98] Fadare JO, Adeoti AO, Desalu OO (2016). The prescribing of generic medicines in Nigeria: knowledge, perceptions and attitudes of physicians. Expert Rev Pharmacoecon Outcomes Res.

[99] (2022). World Health Organization. Launch of the Lomé Initiative. https://www.who.int/dg/speeches/detail/launch-of-the-lom%C3%A9-initiative.

[100] Jairoun AA, Al Hemyari SS, Abdulla NM (2022). Development and validation of a tool to improve community pharmacists’ surveillance role in the safe dispensing of herbal supplements. Front Pharmacol.

[101] Sholy L, Gard P, Williams S, MacAdam A (2018). Pharmacist awareness and views towards counterfeit medicine in Lebanon. Int J Pharm Pract.

[102] Bashir A, Galal S, Ramadan A, Wahdan A, El-Khordagui L (2020). Community pharmacists' perceptions, awareness and practices regarding counterfeit medicines: a cross-sectional survey in Alexandria, Egypt. East Mediterr Health J.

[103] Cheng Y, Liu J, Tan X (2021). Direct comparison of the efficacy and safety of vonoprazan versus proton-pump inhibitors for gastroesophageal reflux disease: a systematic review and meta-analysis. Dig Dis Sci.

[104] Miwa H, Igarashi A, Teng L, Uda A, Deguchi H, Tango T (2019). Systematic review with network meta-analysis: indirect comparison of the efficacy of vonoprazan and proton-pump inhibitors for maintenance treatment of gastroesophageal reflux disease. J Gastroenterol.

[105] Slater BJ, Dirks RC, McKinley SK (2021). SAGES guidelines for the surgical treatment of gastroesophageal reflux (GERD). Surg Endosc.

[106] McKinley SK, Dirks RC, Walsh D (2021). Surgical treatment of GERD: systematic review and meta-analysis. Surg Endosc.

[107] Rickenbacher N, Kötter T, Kochen MM, Scherer M, Blozik E (2014). Fundoplication versus medical management of gastroesophageal reflux disease: systematic review and meta-analysis. Surg Endosc.

[108] Xie P, Yan J, Ye L, Wang C, Li Y, Chen Y, Li G (2021). Efficacy of different endoscopic treatments in patients with gastroesophageal reflux disease: a systematic review and network meta-analysis. Surg Endosc.

[109] Shafie M, Eyasu M, Muzeyin K, Worku Y, Martín-Aragón S (2018). Prevalence and determinants of self-medication practice among selected households in Addis Ababa community. PLoS One.

[110] Divya M BS, Vasudeva G, Varalakshmi C (2016). Self-medication among adults in urban Udupi taluk, southern India. Int J Med Public Health.

[111] Kamati M, Godman B, Kibuule D (2019). Prevalence of self-medication for acute respiratory infections in young children in Namibia: findings and implications. J Res Pharm Pract.

[112] Lee CH, Chang FC, Hsu SD, Chi HY, Huang LJ, Yeh MK (2017). Inappropriate self-medication among adolescents and its association with lower medication literacy and substance use. PLoS One.

[113] White WB, Kloner RA, Angiolillo DJ, Davidson MH (2018). Cardiorenal safety of OTC analgesics. J Cardiovasc Pharmacol Ther.

[114] McCrae JC, Morrison EE, MacIntyre IM, Dear JW, Webb DJ (2018). Long-term adverse effects of paracetamol - a review. Br J Clin Pharmacol.

[115] MacBride-Stewart S, McTaggart S, Kurdi A (2021). Initiatives and reforms across Scotland in recent years to improve prescribing; findings and global implications of drug prescriptions. Int J Clin Exp Med.

[116] Mares-García E, Palazón-Bru A, Martínez-Martín Á, Folgado-de la Rosa DM, Pereira-Expósito A, Gil-Guillén VF (2017). Non-guideline-recommended prescribing of proton pump inhibitors in the general population. Curr Med Res Opin.

[117] Savarino V, Tosetti C, Benedetto E, Compare D, Nardone G (2018). Appropriateness in prescribing PPIs: a position paper of the Italian Society of Gastroenterology (SIGE) - Study section "Digestive Diseases in Primary Care". Dig Liver Dis.

[118] Godman B, Fadare J (2017). Non-guideline-recommended prescribing of proton pump inhibitors: implications for the future and reducing over usage. Curr Med Res Opin.

[119] Siau K, Chapman W, Sharma N, Tripathi D, Iqbal T, Bhala N (2017). Management of acute upper gastrointestinal bleeding: an update for the general physician. J R Coll Physicians Edinb.

[120] Pirmohamed M, James S, Meakin S (2004). Adverse drug reactions as cause of admission to hospital: prospective analysis of 18 820 patients. BMJ.

[121] Chan FK, Graham DY (2004). Review article: prevention of non-steroidal anti-inflammatory drug gastrointestinal complications--review and recommendations based on risk assessment. Aliment Pharmacol Ther.

[122] Rankin A, Cadogan CA, Patterson SM (2018). Interventions to improve the appropriate use of polypharmacy for older people. Cochrane Database Syst Rev.

[123] Marković-Peković V, Škrbić R, Petrović A (2016). Polypharmacy among the elderly in the Republic of Srpska: extent and implications for the future. Expert Rev Pharmacoecon Outcomes Res.

[124] Terblanche A, Meyer JC, Godman B, Summers RS (2017). Knowledge, attitudes and perspective on adverse drug reaction reporting in a public sector hospital in South Africa: baseline analysis. Hosp Pract (1995).

[125] Montané E, Santesmases J (2020). Adverse drug reactions. Med Clin (Barc).

[126] Formica D, Sultana J, Cutroneo PM (2018). The economic burden of preventable adverse drug reactions: a systematic review of observational studies. Expert Opin Drug Saf.

[127] Klatte DC, Gasparini A, Xu H (2017). Association between proton pump inhibitor use and risk of progression of chronic kidney disease. Gastroenterology.

[128] Guedes JV, Aquino JA, Castro TL, Augusto de Morais F, Baldoni AO, Belo VS, Otoni A (2020). Omeprazole use and risk of chronic kidney disease evolution. PLoS One.

[129] Jacob L, Hadji P, Kostev K (2016). The use of proton pump inhibitors is positively associated with osteoporosis in postmenopausal women in Germany. Climacteric.

[130] Jaynes M, Kumar AB (2019). The risks of long-term use of proton pump inhibitors: a critical review. Ther Adv Drug Saf.

[131] Nassar Y, Richter S (2018). Proton-pump inhibitor use and fracture risk: an updated systematic review and meta-analysis. J Bone Metab.

[132] Alghanim SA (2011). Self-medication practice among patients in a public health care system. East Mediterr Health J.

[133] (2015). Medicines update extra. Oral proton pump inhibitors. http://www.ggcprescribing.org.uk/media/uploads/ps_extra/mu_extra_04_-_2015.pdf.

[134] Sun S, Cui Z, Zhou M (2017). Proton pump inhibitor monotherapy and the risk of cardiovascular events in patients with gastro-esophageal reflux disease: a meta-analysis. Neurogastroenterol Motil.

[135] Li S, Liu F, Chen C (2019). Real-world relationship between proton pump inhibitors and Cerebro-cardiovascular outcomes independent of clopidogrel. Int Heart J.

[136] Ben-Eltriki M, Green CJ, Maclure M, Musini V, Bassett KL, Wright JM (2020). Do proton pump inhibitors increase mortality? A systematic review and in-depth analysis of the evidence. Pharmacol Res Perspect.

[137] Melloni C, Washam JB, Jones WS (2015). Conflicting results between randomized trials and observational studies on the impact of proton pump inhibitors on cardiovascular events when coadministered with dual antiplatelet therapy: systematic review. Circ Cardiovasc Qual Outcomes.

[138] Shamliyan TA, Middleton M, Borst C (2017). Patient-centered outcomes with concomitant use of proton pump inhibitors and other drugs. Clin Ther.

[139] Almario CV, Chey WD, Spiegel BM (2020). Increased risk of COVID-19 among users of proton pump inhibitors. Am J Gastroenterol.

[140] Leonard J, Marshall JK, Moayyedi P (2007). Systematic review of the risk of enteric infection in patients taking acid suppression. Am J Gastroenterol.

[141] Vilcu AM, Sabatte L, Blanchon T (2019). Association between acute gastroenteritis and continuous use of proton pump inhibitors during winter periods of highest circulation of enteric viruses. JAMA Netw Open.

[142] Trifan A, Stanciu C, Girleanu I (2017). Proton pump inhibitors therapy and risk of Clostridium difficile infection: systematic review and meta-analysis. World J Gastroenterol.

[143] Lothian Joint Formulary (2021). Gastro-oesophageal reflux disease (GORD). https://formulary.nhs.scot/east/gastro-intestinal-system/gastro-oesophageal-disorders/gastro-oesophageal-reflux-disease-gord/?m=treatment-of-gastro-oesophageal-reflux-disease-gord.

[144] Mohammed I, Nauman A, Paul P (2022). The efficacy and effectiveness of the COVID-19 vaccines in reducing infection, severity, hospitalization, and mortality: a systematic review. Hum Vaccin Immunother.

[145] Wang K, Wang L, Li M (2022). Real-word effectiveness of global COVID-19 vaccines against SARS-CoV-2 variants: a systematic review and meta-analysis. Front Med (Lausanne).

[146] (2003). Supporting prescribing in general practice - a progress report. June.

[147] (2017). National therapeutic indicators and additional prescribing measures 2016/2017. https://www.therapeutics.scot.nhs.uk/wp-content/uploads/2017/07/NTI-17-18-Early-release-document-v1.0.pdf.

[148] Campbell SM, Meyer J, Godman B (2021). Why compliance to National Prescribing Guidelines is important especially across sub-Saharan Africa and suggestions for the future. Biomed Pharm Sci.

[149] Niaz Q, Godman B, Massele A, Campbell S, Kurdi A, Kagoya HR, Kibuule D (2019). Validity of World Health Organisation prescribing indicators in Namibia's primary healthcare: findings and implications. Int J Qual Health Care.

[150] Francke AL, Smit MC, de Veer AJ, Mistiaen P (2008). Factors influencing the implementation of clinical guidelines for health care professionals: a systematic meta-review. BMC Med Inform Decis Mak.

[151] Jankovic DS, Milenkovic AM, Djordjevic AI (2020). Improving the concept of medication vending machine in the light of COVID-19 and other pandemics, 2020 55th International scientific conference on information, communication and energy systems and technologies. ICEST.

